# Cognitive, Language, and Behavioral Outcomes in Children With Autism Spectrum Disorders Exposed to Early Comprehensive Treatment Models: A Meta-Analysis and Meta-Regression

**DOI:** 10.3389/fpsyt.2021.691148

**Published:** 2021-07-26

**Authors:** Bijun Shi, Weijia Wu, Meixia Dai, Jingjing Zeng, Jingyin Luo, Li Cai, Bin Wan, Jin Jing

**Affiliations:** ^1^Department of Maternal and Child Health, School of Public Health, Sun Yat-sen University, Guangzhou, China; ^2^Department of Pediatrics, The Third Affiliated Hospital of Guangzhou Medical University, Guangzhou, China; ^3^Department of Scientific Research, Hainan Women and Children's Medical Center, Haikou, China; ^4^Department of Children's Healthcare and Mental Health Center, Shenzhen Children's Hospital, Shenzhen, China; ^5^Children's Health Care, Hainan Women and Children's Medical Center, Haikou, China; ^6^Max Planck Institute for Human Cognitive and Brain Sciences, Leipzig, Germany

**Keywords:** autism spectrum disorders, comprehensive treatment models, EIBI, ESDM, outcomes, childhood, meta-analysis

## Abstract

**Background:** Early comprehensive treatment models (CTMs) have been developed as effective treatments for children with autism spectrum disorder (ASD). Numerous studies have suggested that CTMs can improve short-term outcomes, but little is known about precise outcome information in childhood. The current meta-analysis reviewed studies reporting broader outcomes in children with ASD who had ever participated in a CTM and examined the predictors of developmental gains.

**Methods:** We searched eight databases up to June 13, 2019, for relevant trials and natural experiments. Longitudinal studies were selected if they investigated the outcomes of CTMs. Two meta-analyses were undertaken to provide a summary estimate of change in treatment outcomes and to evaluate the effect of CTMs; one used the standardized mean change between the pretest and posttest, and the other was a classical meta-analysis. Stratified and random-effects meta-regression analyses were performed to search for outcome differences among studies.

**Results:** Eighteen intervention studies (involving 495 children with ASD) met all the inclusion criteria: 12 used early intensive behavioral intervention (EIBI), and two used the Early Start Denver Model (ESDM). Outcomes were categorized into three parts: cognitive, language and behavioral (e.g., adaptive functioning and symptomatology). Overall, most children with ASD who had ever participated in an early CTM made gains in many areas of functioning, especially in terms of symptom- and language-related outcomes. Stratified analyses indicated that the ESDM displayed the largest effect on IQ improvement (ES = 1.37, 95% CI: 0.95 to 1.80), while EIBI was more effective for symptom reduction (ES = −1.27, 95% CI: −1.96 to −0.58). Further, meta-regression suggested that interventions with parent involvement, higher intensity, and longer treatment hours yielded greater improvements in IQ and social adaptive functioning, respectively.

**Conclusion:** The results demonstrate a positive association between CTMs and better prognosis in childhood, especially regarding symptoms, and language. However, most extant research involves small, non-randomized studies, preventing definitive conclusions from being drawn. Clearly, the outcomes of children with ASD are still far from normal, especially with respect to adaptive functioning, and the four mediating variables pertaining to treatment elements can affect their gains, including approach, implementer, intensity, and total treatment hours.

**Systematic Review Registration:** [www.crd.york.ac.uk/PROSPERO], identifier [CRD42019146859].

## Background

Autism spectrum disorder (ASD) is a common neurodevelopmental disorder characterized by persistent impairment in social communication and repetitive, restricted patterns of behaviors and interests (1–3); it affects 1–2% of children (4, 5) and usually has a serious influence on development and lifetime costs (6). Behavioral treatments are considered as the recommended therapies to treat symptoms of ASD (7). As therapy progresses, it has moved from isolated teaching episodes toward teaching in the natural environment. Besides, a growing number of interventions are informed by child development theories (8).

Many behavioral interventions, particularly for young children with ASD, have shown positive effects on cognition, language functioning, and core symptoms (9, 10); in most cases, only immediate outcomes at the end of the intervention or during the first 5 years of life were reported (11, 12). However, even significant improvements in short-term outcomes do not fully establish treatment effectiveness because developmental gains could diminish after intensive services end (13). Two narrative reviews that sought to clarify the long-term effects were limited due to the small number and poor quality of eligible follow-up studies (14, 15). Robust studies on novel comprehensive treatment models (CTMs), such as Learning Experiences - An Alternative Program for Preschoolers and Parents (LEAP), are regarded as the key to long-term efficacy (7). Thus, more subsequent trials in this field should be replicated and validated in different countries in the future.

It is likely that the increase in functional skills (i.e., intelligence) that allows children to gain more from later experiences is a long-term mediating mechanism allowing them to maintain gains (16), highlighting the importance of outcomes in each postintervention period. Most existing systematic reviews focused on the effect of early autism interventions and involved mainly the outcomes in preschool children (17, 18). However, there is limited understanding of outcomes post-middle childhood (i.e., 5 years and later) (19). Moreover, the existing findings regarding mid-childhood cognitive ability and adaptive functioning outcomes in children with ASD have shown considerable variability. For example, Magiati et al. (20) reported negative outcomes on children aged 10 years, but Este et al. (13) reported the opposite results in children aged 6 years. In addition, a comprehensive collaboration among the families, the intervention team, and the receiving teachers as well schools is frequently lacking during the young children's transition to school. A recent meta-analysis indicated that almost half of individuals with ASD had poor outcomes in later adolescence and adulthood (21). However, we still lack any secondary research evidence focused explicitly on the outcomes in 5–18-year-old children. Increasing our understanding of outcomes in childhood is helpful to enact effective school curriculum and targeted support.

In addition to understanding the outcomes, it is also important to identify the factors influencing developmental gains, which can help to explain the heterogeneity across the studies and inform the establishment of intervention strategies. A small amount of evidence indicates that children's pretreatment levels and treatment elements may affect the efficacy of treatment (22, 23), raising questions about the predictors of developmental gains for children. Both of the more well-established CTMs for ASD, referred to as early intensive behavioral intervention (EIBI) and the Early Start Denver Model (ESDM), are rooted in principles of applied behavior analysis (ABA). However, ESDM is also a parent-involvement, relationship-based intervention that fuses approaches validated by the science of child development, and there are few comparative evaluations of different programs (11). If intervention approaches play a role, this role should not be underestimated. Thus, given that the transition to school and community is often difficult and stressful for individuals with ASD and their families, there is a pressing need for systematic knowledge of the outcomes in childhood and their predictive factors in children with ASD who have been exposed to a CTM to provide timely support (24).

Above all, the present study aims to extend previous reviews by conducting a meta-analysis and meta-regression of longitudinal studies from early childhood to adolescence. The study aimed to (1) report outcomes for specific domains of functioning and behavior (including cognition, language, adaptive functioning and symptomatology); (2) discover whether there are significant improvements in those outcomes for children with ASD and the effect of the CTMs; and (3) examine the influence of childhood pretreatment characteristics, study characteristics, and intervention elements on gains.

## Methods

The protocol for this meta-analysis was registered in the PROSPERO database of prospectively registered systematic reviews (www.crd.york.ac.uk/PROSPERO; CRD42019146859), and the completed study conforms to the guidelines of the Preferred Reporting Items for Systematic Reviews and Meta-Analyses (25).

### Search Strategy and Selection Criteria

A systematic literature search was performed in eight electronic databases: PubMed, EMBASE, PsycINFO, Scopus, the Cochrane Library, OVID, ERIC, and Web of Science. Each database was initially searched for relevant literature in English from its inception through June 13, 2019. We developed a search strategy for PubMed based on MeSH (Medical Subject Headings) terms and text words from key research that we identified a priori (see Supplementary Table 1 for the full search strings). We reviewed the reference lists of key publications and relevant narrative reviews to identify studies that might have been missed in the database searches. To check for possible publication bias, we also undertook a gray literature search in clinical trial registries (http://www.ClinicalTrials.gov) using identical inclusion criteria to identify unpublished trials.

After the removal of duplicates, two independent investigators performed title scans and abstract reviews, and they screened the full-text articles to assess their eligibility for inclusion. Concordance among the investigators was satisfactory, with a positive agreement of 0.83; any disagreements between the authors were resolved by consultation with the third investigator. A number of prespecified inclusion and exclusion criteria were used to select key studies. The inclusion criteria were as follows: (a) randomized controlled trials (RCTs), quasi-experimental studies (i.e., non-equivalent control group design, one-group pretest/posttest design), and natural experiments (a form of observational study in which the researcher cannot control or withhold the allocation of an intervention to particular areas or communities; thus, natural or predetermined variation in allocation occurs); (b) longitudinal studies with at least one assessment in early childhood and one in mid-childhood or adolescence; (c) mean age of participants at first assessment (“early childhood”) <5 years; (d) mean age of participants at last assessment (“mid-childhood or adolescence”) between 5 and 18 years; (e) professional/clinical diagnosis of ASD, autism, PDD-NOS, or Asperger syndrome based on DSM criteria; (f) English-language articles published in a peer-reviewed journal (dissertations were excluded); and (g) articles assessing the effectiveness of a CTM and reporting primary outcome variables focused on child functioning.

The following exclusion criteria were applied: (a) studies including children with medical complications or who were receiving drug treatment; (b) pharmacological or dietary interventions, focused intervention practices [FIP, e.g., Pre-school Autism Communication Trial (PACT), Joint Attention, Symbolic Play and Engagement Regulation (JASPER)], and other interventions with unclear evidence according to National Institute for Health and Care Excellence (NICE) guidance, such as secretin, chelation, or hyperbaric oxygen therapy; (c) studies reporting on a CTM that was not present in at least two other studies, that is, “isolated intervention approaches”; and (d) studies for which pre- and posttest means and standard deviations were not available after attempts to contact the authors and could not be calculated from the descriptive data or statistical tests in the study manuscript. For multiple studies on the same cohort, we selected the publication with the longest follow-up, provided it included results with detailed demographic and intervention information.

### Data Extraction and Quality Assessment of the Included Studies

Pairs of investigators independently performed data extraction with a predesigned standardized form, and discrepancies were resolved by repeated discussion until consensus was reached. To ensure the accuracy and completeness of the extracted information, the third investigator repeatedly verified the extracted data abstraction for all the included studies. The following information from each included study was extracted: first author; region, study design, and year of publication; population characteristics at intake, including subtype of sample, age, and sex (% male); intervention characteristics, including intervention approaches (e.g., EIBI, ESDM), setting (clinical/home), implementer (therapists/therapists and parents), intensity and duration in weeks and months; type of comparison (e.g., treatment as usual, implementer, intensity, and no comparison group); assessment times (i.e., pre, post, follow-up); the measures employed in each study; and the outcomes reported in childhood (e.g., autism symptomatology, IQ, adaptive behavior, language).

Two independent investigators applied the Evaluative Method for Determining Evidence-Based Practices in Autism to assess the quality of the included studies (26), which is available for many study designs. A previous study suggested that this tool can be applied to evaluate intervention studies and produce valid assessments of the empirical evidence on practices in children with ASD (27). Six primary and eight secondary quality indicators were applied and are annotated in Supplementary Table 3, including the characteristics of the participants, independent variables, dependent variables, comparison conditions, random assignment, blinding of raters, and fidelity. Divergence between the two investigators who evaluated the quality of the studies was resolved by discussion. The quality of a study was assessed as “strong” when all the primary indicators received high quality ratings and there were four or more secondary indicators; “adequate” when more than four primary indicators received high ratings, with no unacceptable ratings and evidence of at least two secondary indicators; and “weak” otherwise.

### Calculation of Effect Sizes

Because the instruments for evaluating a given outcome differed across studies (e.g., Wechsler Intelligence Scale for Children vs. Merrill-Palmer Scales of Mental Tests), we used standardized ESs to obtain standardized measurements of the effect of the intervention on the outcome variables. According to the methodology of Reichow and Wolery (28), two types of ES were computed: the standardized mean change ES (g_c_) and the standardized mean difference (SMD) ES (g_d_). We took two steps to ensure the most conservative ES. First, ESs were calculated only when the data necessary for the calculation were available. If an outcome variable was missing the necessary data for the calculation of an ES, no ES was calculated for that outcome of the study. Hence, no data were extrapolated or interpolated for the calculation of ESs. Second, ESs based on small samples are known to be biased (29), so we multiplied them by the small sample correction factor (30).

The first ES analyses were calculated for the intervention groups in all the included studies and examined the differences between the average gains made by distinct samples. This comparison revealed the absolute difference within a sample from preintervention to childhood without regard to the comparison group in between-group studies. We calculated the g_c_ by dividing each adjusted mean change by the pooled standard deviation.

For the between-group studies, the g_d_ was used to show the magnitude of the difference between the group receiving a CTM and the comparison group. The ES (g_d_) was calculated by dividing each adjusted mean difference by the pooled standard deviation.

### Meta-Analytic Procedures

We combined findings from all the included studies using prespecified meta-analytic methods to determine the effect of CTMs in children with ASD. Data synthesis involved two steps: (1) Meta-analysis I was performed to estimate longitudinal changes in broader outcomes in children with ASD who were exposed to a CTM. (2) Meta-analysis II was performed to assess the effect of CTMs on those outcomes in the test group compared to the control group. The standardized mean change/difference and 95% confidence interval (CI) for each intervention effect were the primary outcome measures in the meta-analysis. Due to the diversity in population characteristics and intervention approaches, we expected a conservative estimation of the ESs. Consequently, a meta-analysis was performed on studies judged sufficiently similar and appropriate to pool using random effects models. Cohen's criteria (31) were applied to determine the magnitude of the effect. The magnitude of the effect was assessed as “trivial” when the ES was <0.2, “small” when the ES was between 0.2 and 0.49, “medium” when the ES was between 0.5 and 0.79, and “large” when the ES was ≥0.8.

Prespecified and exploratory stratified analyses were conducted to assess differences in ESs based on the use of (1) EIBI, (2) ESDM, and (3) other interventions to examine the consistency of the intervention approaches. Outcomes reported in fewer than six studies and parental outcomes were discarded from the meta-analysis, and studies were rank-ordered by quality rating in the forest plots.

The *I*^2^ statistic was used to assess the potential heterogeneity of ESs across interventions. An *I*^2^ >50% was considered evidence of heterogeneity. Potential publication bias was assessed in two ways: a funnel plot and Egger's linear regression test. When publication bias was identified, a non-parametric trim-and-fill method was used to adjust for the publication bias. Sensitivity analysis was performed by reanalyzing the data using a fixed effects model and by omitting one study at a time to assess the impact of each individual study on the overall pooled estimate. Moreover, we re-ran all the Meta-analysis I models restricting the study design to the between-group controlled studies.

### Meta-Regression

Although, there are certainly variations across the included studies (i.e., varying amounts of time between posttreatment and the collection of follow-up data, total treatment hours), we applied random-effects meta-regression analyses to examine the effect of moderators and mediators on primary outcomes and to explore the potential heterogeneity. A moderator (baseline variable) suggests for whom or under what conditions a treatment might affect the outcome of interest. A mediator (intervention variable) suggests how or why the treatment might work. Three categories were defined a priori in the protocol: (1) internal validity (risk of bias, sample size), (2) population characteristics (preintervention age, preintervention IQ, time interval between postintervention and follow-up, age at the last assessment), and (3) intervention characteristics (intervention approaches, total intervention hours [duration multiplied by intensity], intensity (hours/week), duration (months), implementer [therapist, therapist and parent]). To reduce the risk of type II errors, we abstained from performing regression with predictors that were available for <6 of the included trials, and univariate meta-regression was used for predictors available in 6–10 of the included trials. Only for IQ, which was reported in >10 trials, were all variables that predicted variance (*p* < 0.05) included in a multivariate regression model, and forward elimination was performed. Given the type I errors of the multivariate meta-regressions, we also applied the Monte Carlo permutation test. Besides, we performed some binary meta-regression plots to evaluate the linear relationship between intervention characteristics and the primary outcome measures.

All meta-analytic procedures were performed with STATA 12.0 (Stata Corp., College Station, TX, USA).

## Results

### Literature Search and Study Characteristics

**Figure 1:**
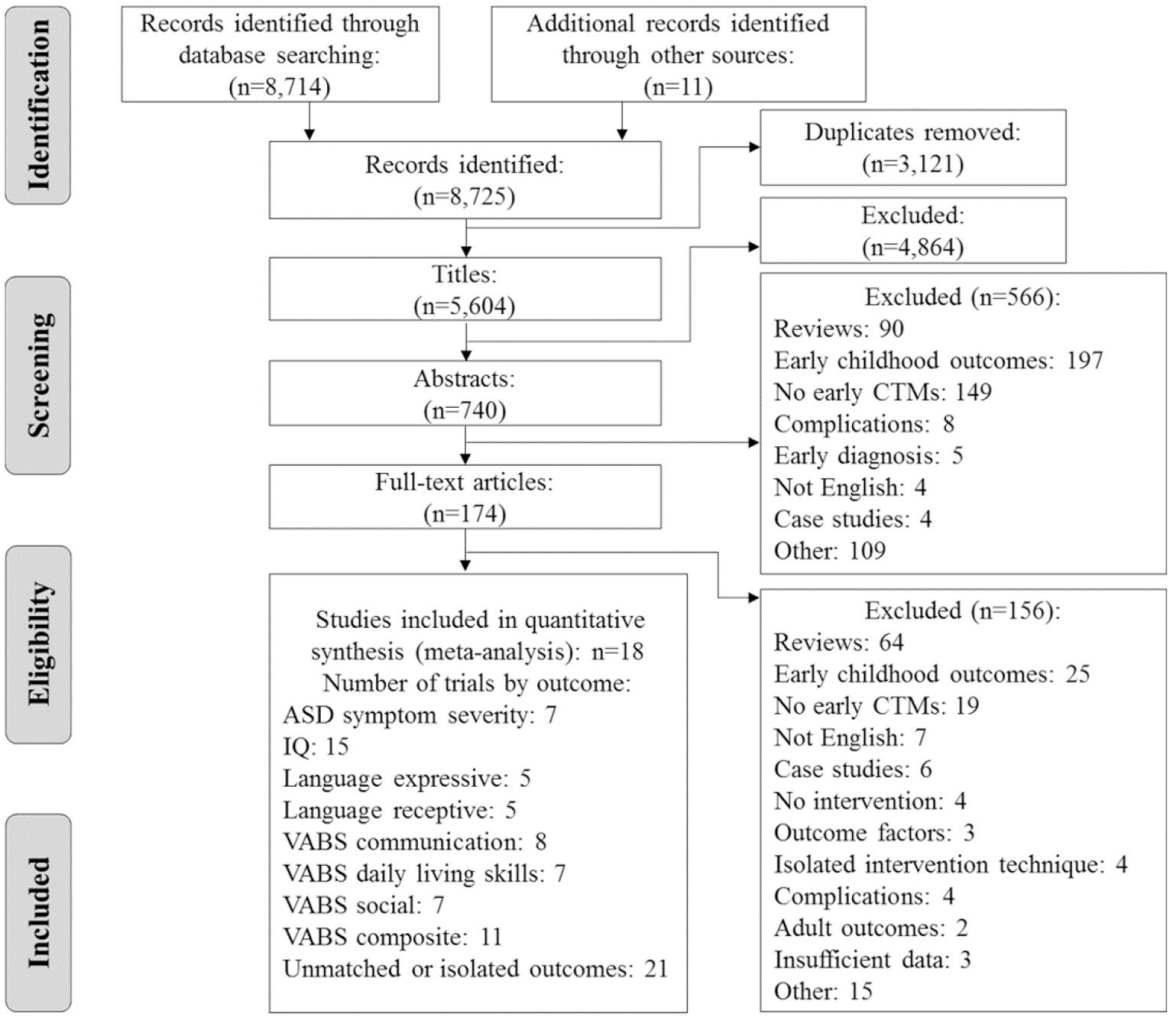
Flowchart of the retrieval and selection of references. ASD, autism spectrum disorder; CTM, comprehensive treatment models; IQ, intelligence quotient; VABS, Vineland Adaptive Behavioral Scales.

A flow diagram detailing the selection process is presented in Figure 1. We identified 8,725 potentially relevant citations, and 174 full citations were retrieved. Two reports, Lovaas (32) and McEachin et al. (33), used the same participants. The latter was selected because it has the longest follow-up. Overall, 18 unique citations were deemed eligible for the systematic review and meta-analysis (13, 20, 33–48).

A systematic description of eight between-group studies and 10 prepost studies (including 495 non-overlapping participants with ASD) is provided in Table 1. Three of the 10 prepost studies with within-subject designs were natural experiments, and the intervention characteristics were reported by parents. Half of the included studies were postintervention follow-ups and thus had a period of time during which the intervention was not being implemented; the outcomes from these studies were defined as “long-term.” These studies used a wide range of measures to assess autism symptom severity, cognitive and language abilities, and adaptive behavior (Supplementary Table 2). Most employed standardized measures and researcher-developed interviews, and all the repeatedly measured outcomes were standard scores. Moreover, six studies (33%) received the highest rating (strong), two (11%) received the middle rating (adequate), and 10 studies (56%) received the lowest rating (weak; Supplementary Table 3) based on the assessment of research report rigor.

**Table 1:** Characteristics of the studies included in the meta-analysis reporting multiple outcomes in children with ASD.

**Study**	**Region**	**Design**	**Participants**				**Intervention characteristics^c^**					**Control group**	**Rigor rating^d^**
			**Sample^a^ (*n*, male%)**	**Diagnosis (criteria)**	**Pre-test CA^b^ (months)**	**Pre-IQ**	**Methods (model)**	**Intensity (h/week)**	**Duration (months)**	**agent**	**Post-test/follow-up CA (months)**		
Akshoomoff et al. (34)^#^	USA	Pre-post experimental	20 (90.00%)	ADPDD-NOS (DSM-IV)	28.90 (2.70)	—	others^e^	31.00	7.70 (2.20)	T + P	85.30 (27.80)	NO	Weak
Bibby et al. (35)	UK	Pre-post observational	22 (83.33%^‡^) 21^f^	ASDPDD	45.00 (11.20)	50.80 (20.60)	EIBI (UCLA)	30.30 (5.50)	31.60 (11.90) 33.20^f^	T	77.40 (15.00) 78.70^f^	NO	Weak
Clark et al. (36)	AUS	Pre-post observational	48 (75%)	ADASD (DSM-IV)	25.45 (2.12)	65.68 (11.87)	others	NR	NR	T	96.50 (6.60)	NO	Weak
Cohen et al. (37)^g^	USA	Between-group NRT	21 (85.71%)	ADPDD-NOS	30.20 (5.80)	61.60 (16.40)	EIBI (UCLA)	35–40	36.00	T + P	66.24 (5.76)	YESN-R	Strong
Estes et al. (13)^#^	USA	Between-group RCT	21	ADPDD-NOS (DSM-IVTR)	23.90 (4.00)	61.00^h^ (9.20)	ESDM	31.50	24.00	T + P	72.90 (2.60)	YESRandom	Strong
Gabriels et al. (38)	USA	Pre-post^i^ observational	17 (70.59%)	AutismPDD-NOS	30.60 (7.27)	57.81 (25.88)	others	22.63	36.00	T	68.70 (10.11)	NO	Weak
Harris et al. (39)	USA	Pre-post experimental	27 (85.19%)	AD (DSM-III-R)	49.00 (31-65)	59.33 (23.75)	EIBI	35–45	36.00	T + P	85.00	NO	Weak
Howard et al. (40)	USA	Between-group NRSI observational	29 (86.00%)	ADPDD-NOS (DSM-IV)	30.86 (5.16)	60.57 (17.48)	EIBI (IBT)	35–40	37.90 (2.98)	T + P	69.24 (5.01)	YesN-R	Strong
Kovshoff et al. (47)^#^	UK	Between-group NRT	23	Autism	35.70 (4.00)	61.43 (16.43)	EIBI	25.60 (4.80)	24.00	T + P	83.70	YesN-R	Adequate
Landa and Kalb (41)^#^	USA	Pre-post experimental	48 (81.25%)	ASD	27.20 (2.80)	60.10 (11.90)	others	10.00	6.00	T + P	72.60 (17.50)	No	Weak
McEachin et al. (33)^j^#^^	USA	Between-group NRT	19 (84.21%)	Autism (DSM-III)	34.60	53.00 (30–82)	EIBI (UCLA)	40.00	60.00	T + P	156.00 (108-228)	YESN-R	Strong
Magiati et al. (20)^#^	UK	Pre-post experimental	36	AutismASD	38.90 (7.10)	64.40 (30.00)	EIBI (UCLA)	30.00	57.90 (21.20)	T	123.60 (9.60)	No	Weak
Perry et al. (48)^#^	CA	Pre-post experimental	21 (90.48%)	ADPDD-NOS (DSM-IV)	40.92 (12.60)	—	EIBI	20–40	26.76 (9.84)	T	192.20 (21.48)	No	Weak
Sallows et al. (42)	USA	Between-group RCT	13 (84.61%)	Autism (DSM-IV)	33.23 (3.89)	50.85 (10.57)	EIBI (UCLA)	38.60 (2.91)	48.00	T	83.23 (8.92)	YesRandom	Strong
Smith et al. (43)^#^	USA	Between-group RCT	15 (80.00%)	AutismPDD/NOS	36.07 (6.00)	50.53 (11.18)	EIBI (UCLA)	24.52 (3.69)	33.44 (11.00)	T	94.07 (13.17)	YesRandom	Adequate
Smith et al. (44)^#^	USA	Pre-post experimental	64 (84.51%)^‡^	ASD	39.12 (7.92)	58.80 (13.39)	EIBI (UCLA)	16.66	12.00	T	67.80 (9.72)	No	Weak
Vinen et al. (45)	AUS	Between-group NRSI	31 (87.10%)	ASD (DSM-IV, DSM-V)	39.16 (9.91)	55.42^h^ (8.74)	ESDM	≥15	22.44	T + P	79.97 (7.99)	YesN-R	Strong
Weiss and Delmolino (46)	USA	Pre-post experimental	20 (95.00%)	AutismPDD/NOS (DSM-IV)	41.50 (20–65)	—	EIBI (IBT)	40.00	48.00	T	89.5	No	Weak

a *Total number of subjects at the last measurement for pre-post studies and subjects in the experimental group for between-group studies*.

b *Chronological age at which the participants entered the study or started the intervention*.

c *Intervention characteristics for pre-post studies and the experimental group's features for between-group studies*.

d *The quality assessment was examined by the Evaluative Method for Determining Evidence-Based Practices in Autism (51)*.

e *Others (other interventions) refers to the combination of standard interventions, including discrete trial training, incidental teaching, pivotal response training, structured teaching, and the picture exchange communication system (e.g., community, inclusive intervention)*.

f *The samples are inconsistent between the two outcomes reported by Bibby et al. (35)*1.

g *Sufficient data were acquired from the figures in Cohen et al. (37)*.

h *The early learning composite (ELC) from MSEL was used to report cognition function*.

i *Gabriels et al. (38) was a retrospective case-control study conducted on one sample receiving the same treatment and examined the influencing factors of the best outcomes*.

j *Two reports, Lovaas (32) and McEachin et al. (33), used the same participants. The McEachin et al. (33) report was used because it has the longest follow-up*.

‡ *Male% was not reported in follow-up subjects. We used male% at intake to replace it*.

# *Those included studies were postintervention follow-ups and thus had a period of time during which the intervention was not being implemented*.

*ABA, applied behavior analysis; AD, autism disorder; ASD, autism spectrum disorder; AUS, Australia; CA, chronological age; CA, Canada; DSM, The Diagnostic and Statistical Manual of Mental Disorders; EIBI, early intensive behavioral intervention; ESDM, the Early Start Denver Model; IBT, intensive behavioral treatment; IQ, intelligence quotient; PDD/NOS, pervasive developmental disorder not otherwise specified; T, therapist; T + P, therapist and parents; N-R, non-random; NR, not reported; NRT, non-randomized trial; NRSI, non-randomized study for intervention; RCT, randomized controlled trial; UCLA, University of California, Los Angeles*.

### Population and Intervention Characteristics

The mean pre-IQ, reported in 15 studies, was 50–64; the mean pretest age was 24–49 months, and the mean age at the last assessment was 66–192 months. Of the 18 studies included, 12 conducted EIBI [seven applied the UCLA model (32)], two used the ESDM, and four used other interventions. Other interventions (e.g., community intervention) include the combination of standard interventions. With regard to the intervention characteristics, eight studies were implemented by therapists and parents. The intervention duration and intensity ranged from 6 to 60 months and from 15 to 40 weekly hours, respectively. Six studies reported that participants were receiving supplemental treatments. Moreover, the comparison conditions in the eight between-group studies, which included 6 EIBI programs and 2 ESDM programs, were treatment as usual (k = 5), different implementers (k = 2), and active comparison (k = 1).

### Outcomes and Meta-Analysis I: Longitudinal Change in Childhood

**Figure 2:**
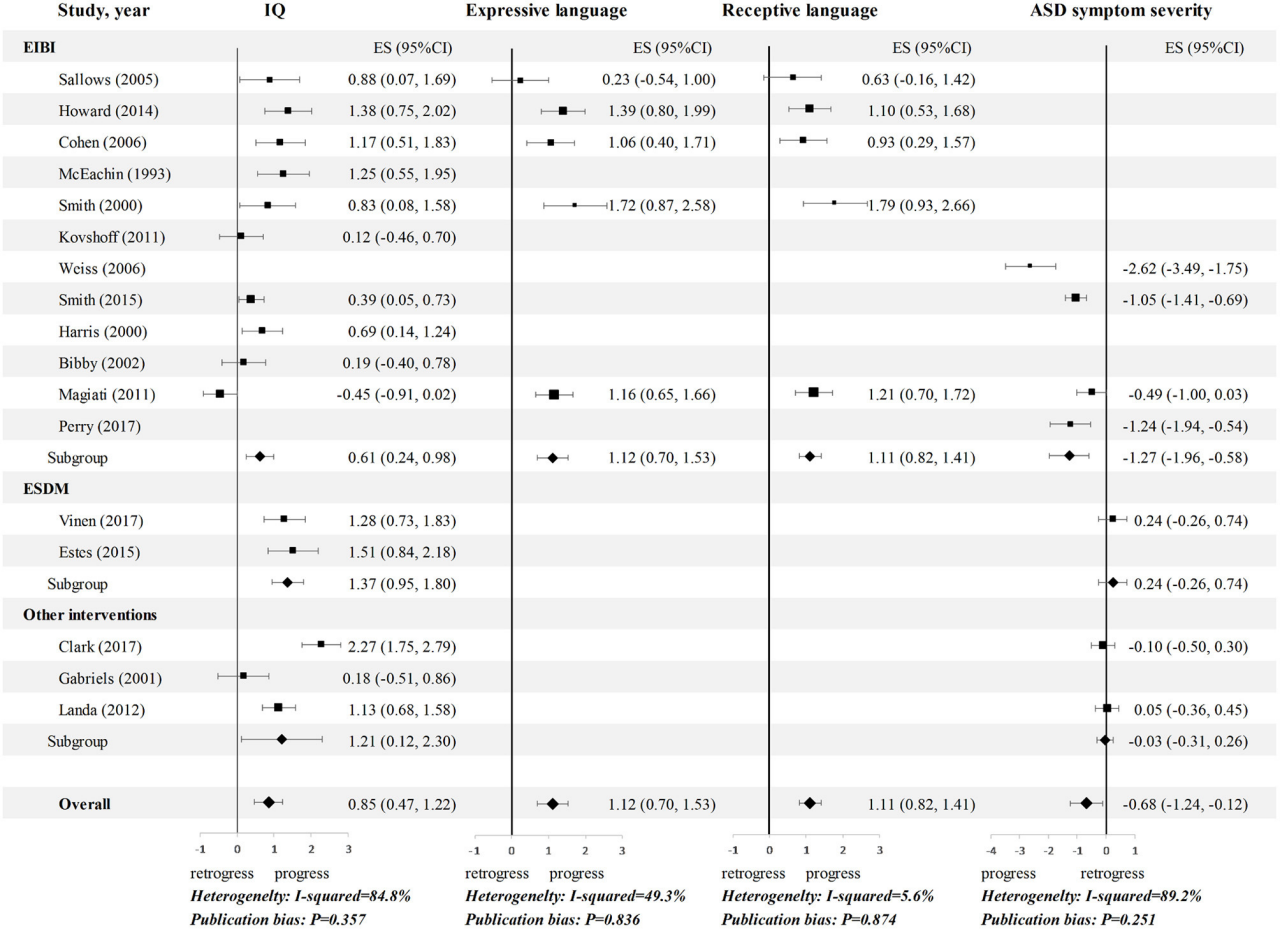
Meta-analysis 1: Effect sizes (gc) for IQ, language and symptom outcomes in children with ASD. Hedges' g effect sizes represented in black and confidence intervals are reported. Random effects models were used on all outcomes, and the studies were rank-ordered by quality rating. ASD, autism spectrum disorder; CI, confidence interval; EIBI, early intensive behavioral intervention; ES, effect sizes; ESDM, Early Start Denver Model; IQ, intelligence quotient.

**Figure 3:**
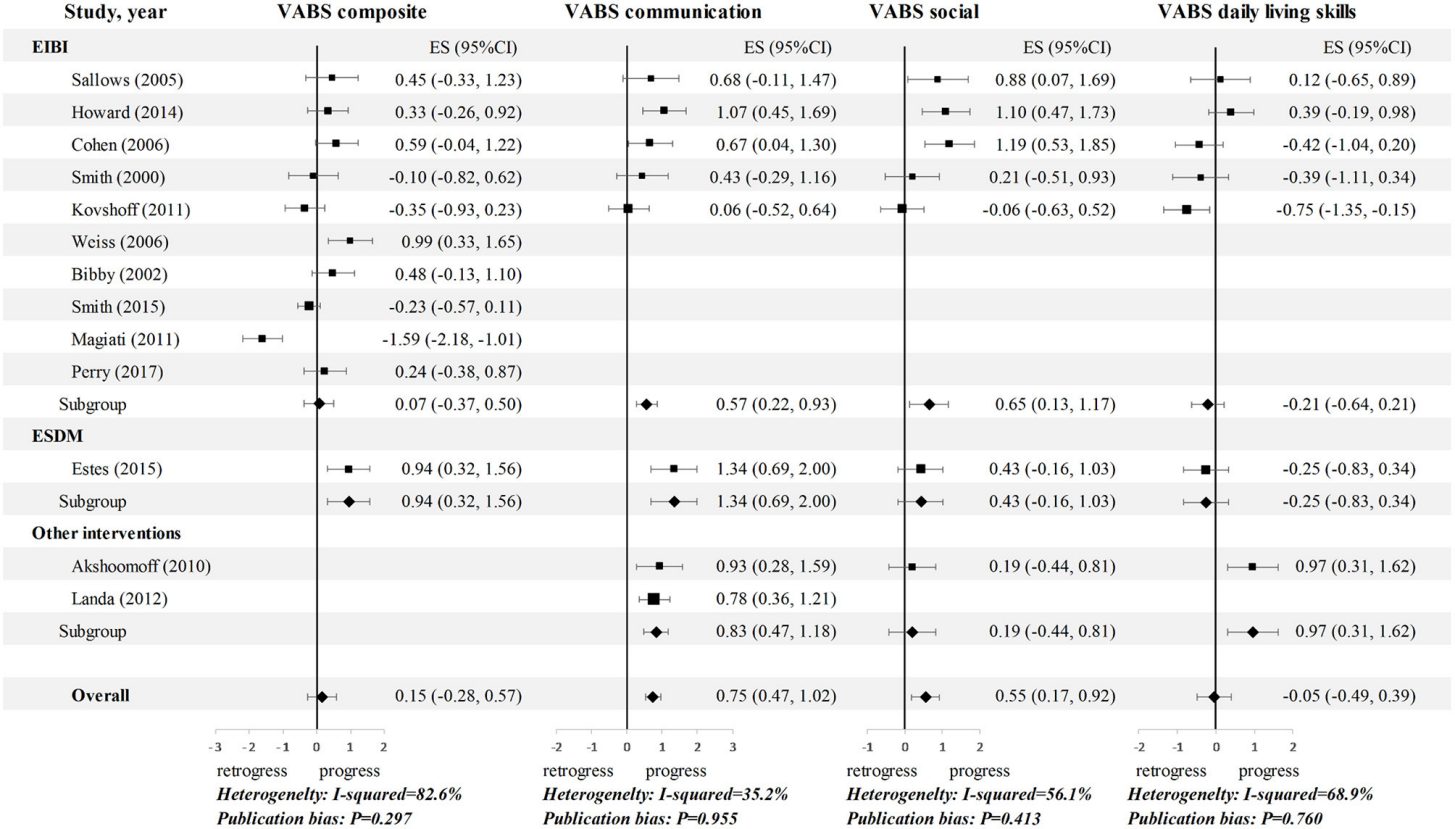
Meta-analysis 1: Effect sizes (gc) for adaptive functioning in children with ASD. Hedges' g effect sizes represented in black and confidence intervals are reported. Random effects models were used on all outcomes, and the studies were rank-ordered by quality rating. CI, confidence interval; EIBI, early intensive behavioral intervention; ES, effect sizes; ESDM, Early Start Denver Model; VABS, Vineland Adaptive Behavioral Scales.

Although, a number of studies evaluated outcomes across multiple domains, others focused on specific areas, such as intellectual abilities, adaptive functioning, language outcomes, or autism severity. A summary of reported outcomes is presented in Table 2; generally, positive ESs (g_c_) suggest that children's performance improved on average after the preintervention stage in multiple dimensions of functioning (see Figures 2, 3).

**Table 2:** Summary of cognitive, language, symptomatic, and adaptive functioning outcomes in childhood.

**Study**	**IQ^d^**		**Expressive language^e^**		**ASD Symptom Severity^f^**		**Adaptation composite^g^**	
	**Preintervention**	**Middle childhood**	**Preintervention**	**Middle childhood**	**Preintervention**	**Middle childhood**	**Preintervention**	**Middle childhood**
Bibby	50.80 ± 20.60	55.00 ± 22.30					54.50 ± 13.00	63.40 ± 21.90
Clark^a^	65.68 ± 11.87	102.71 ± 19.55			6.45 ± 2.08	6.20 ± 2.68		
Cohen^b^	61.60 ± 16.40	87.00 ± 25.26	52.90 ± 14.50	78.00 ± 29.91			69.80 ± 8.10	79.00 ± 19.77
Estes^c^	61.00 ± 9.20	90.52 ± 26.36					69.50 ± 5.70	81.41 ± 17.27
Gabriels	57.81 ± 25.88	62.94 ± 30.79						
Harris	59.33 ± 23.75	77.59 ± 28.10						
Howard	60.57 ± 17.48	89.43 ± 23.99	49.73 ± 16.34	83.25 ± 29.88			72.00 ± 7.73	76.00 ± 15.94
Kovshoff	61.43 ± 16.43	64.65 ± 33.04					60.22 ± 5.82	55.13 ± 19.40
Landa	60.10 ± 11.90	81.50 ± 24.40			7.30 ± 2.20	7.40 ± 2.00		
McEachin^c^	53.00 ± 13.00	84.50 ± 32.40						
Magiati	64.40 ± 30.00	52.60 ± 21.80	2.60 ± 7.30	34.50 ± 37.90	36.70 ± 7.20	32.40 ± 10.00	58.70 ± 5.90	37.20 ± 17.90
Perry					34.16 ± 5.49	26.63 ± 6.40	63.45 ± 8.95	66.85 ± 17.18
Sallows	50.85 ± 10.57	73.08 ± 33.08	47.92 ± 6.17	53.38 ± 31.91			59.54 ± 5.31	69.00 ± 28.04
Smith 2000	50.53 ± 11.18	66.49 ± 24.08	15.13 ± 0.52	44.53 ± 23.48			63.44 ± 9.35	61.19 ± 29.72
Smith 2015	58.80 ± 13.39	64.93 ± 18.01			8.51 ± 1.76	6.45 ± 2.15	62.68 ± 9.02	59.89 ± 14.65
Vinen	55.42 ± 8.74	76.06 ± 20.82			7.39 ± 2.09	7.97 ± 2.60		
Weiss					45.68 ± 5.30	26.58 ± 8.60	49.85 ± 7.84	76.05 ± 36.01

a *Data were acquired from the merging of subgroups in Clark et al. (36)*.

b *Data were acquired from the figures in Cohen et al. (37)*.

c *The standard deviation is calculated from the range of the outcomes in Estes et al. (13) and McEachin et al. (33)*.

d *IQ was measured by a series of instruments, including WISC, BSID, WPPSI, and so on*.

e *Language was measured by Reynell, SICD-R, EOWPVT, and BPVS-2*.

f *ASD symptom severity was measured by ADOS, ADI-R, and CARS*.

g *Adaptation composite was measured by VABS*.

*Akshoomoff et al. (34) reported the subdomains of adaptive functioning and non-verbal/verbal IQ, which are not represented in Table 2. ASD, autism spectrum disorder; IQ, intelligence quotient*.

The pooled standardized mean change ES for IQ, covering 420 participants, was 0.85 (95% CI: 0.47 to 1.22). Only one study (20) had a negative ES for IQ, while 10 of the other samples yielded an ES for IQ equal to or >0.50. Five EIBI studies reported data on language skills, four of which reported favorable effects on both expressive and receptive language. The pooled ESs for expressive language and receptive language were 1.12 (95% CI: 0.70 to 1.53) and 1.11 (95% CI: 0.83 to 1.40), respectively. Regarding the longitudinal changes in ASD symptom severity, seven studies reported relevant data, and three of them showed a favorable effect. The pooled ES was −0.68 (95% CI: −1.24 to −0.12). For adaptive functioning, the subdomains showed heterogeneity (Figure 3). A medium ES was found for both communication (ES = 0.75, 95% CI: 0.47 to 1.02) and social (ES = 0.55; 95% CI: 0.17 to 0.92), whereas, a trivial ES was found for daily living skills (DLS) (ES = −0.05, 95% CI: −0.49 to 0.39) and composite score (ES = 0.15, 95% CI: −0.28 to 0.57).

### Meta-Analysis II: Effects of EIBI on Outcomes in Childhood Compared to Those in the Control Group

**Figure 4:**
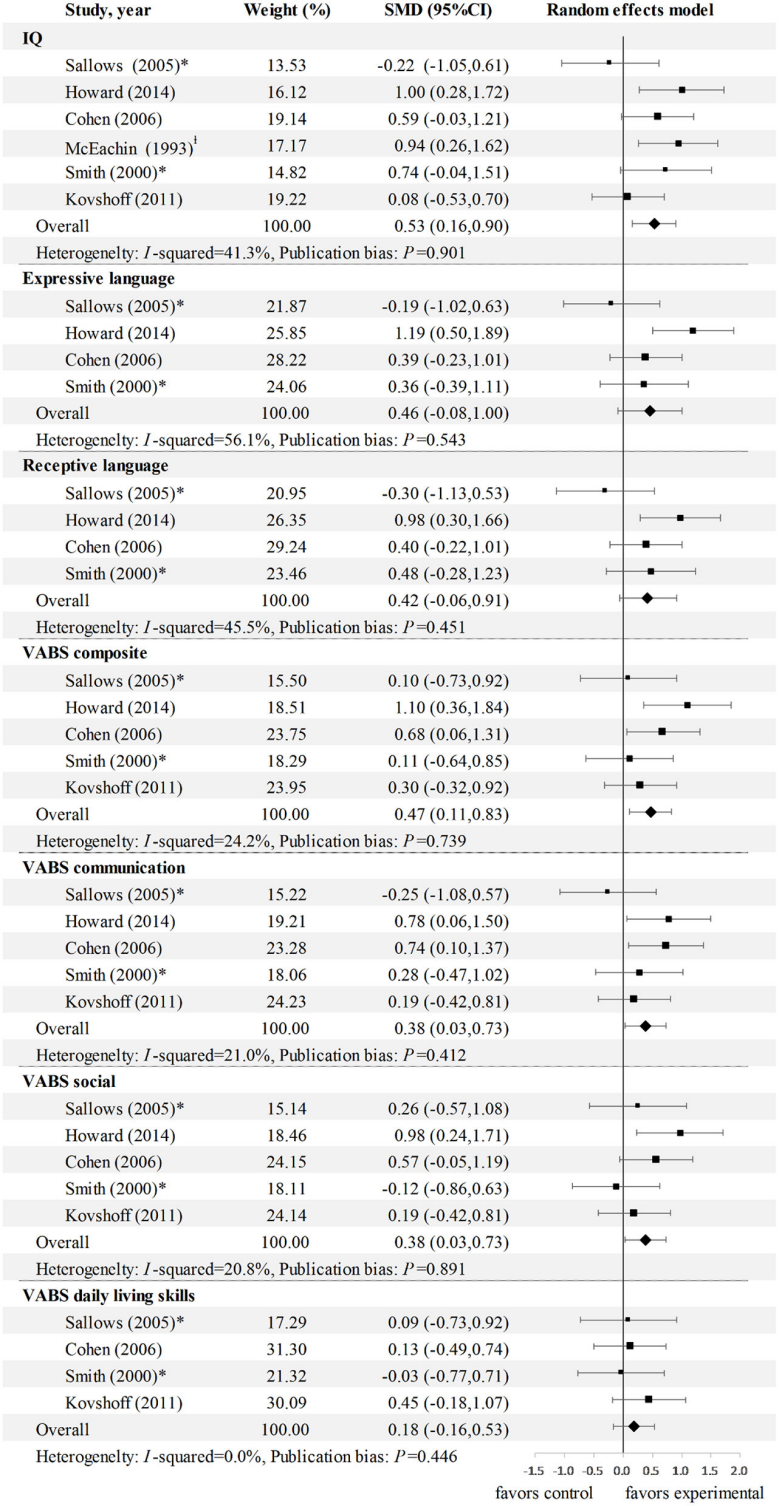
Meta-analysis 2: SMD (gd) for multiple outcomes of EIBI in children with ASD. comparison type * EIBI therapist vs. EIBI parents; l EIBI vs. EIBI minimal intensity. CI, confidence interval; ES, effect sizes; IQ, intelligence quotient; SMD, standardized mean difference.

As presented in Figure 4, the majority of the SMD ESs (g_d_) were positive, which indicates that the functioning of children with ASD in the EIBI group was generally better than that in the comparison group in multiple dimensions. In line with the longitudinal change results, EIBI had small to medium effects in terms of improving IQ (ES = 0.53, 95% CI: 0.16 to 0.90), communication (ES = 0.38, 95% CI: 0.03 to 0.73), and social (ES = 0.38, 95% CI: 0.03 to 0.73). The ES for DLS was also non-significant in four studies (ES = 0.18; 95% CI: −0.16 to 0.53). However, we failed to find a favorable improvement in expressive and receptive language when the analysis was applied solely to controlled studies (ES = 0.46, 0.42; 95% CI: −0.08 to 1.0, −0.06 to 0.91, respectively). Additionally, adaptation composite scores were reported in five studies, resulting in a significant effect size of 0.47 (95% CI 0.11 to 0.83).

The controlled ESDM studies and the outcome for ASD symptom severity were discarded from meta-analysis II because of inadequate or isolated data.

### Stratified Analyses

The results for the comparison of the three intervention approaches in the stratified analyses of meta-analysis I revealed disparate effects. Notably, the ESDM group had a significantly higher ES for IQ than the EIBI and other interventions groups (g_c_ = 1.37, 0.61, and 1.21, respectively; Figure 2). Regarding other outcomes, the number of ESDM studies is insufficient for comparison. Nevertheless, the opposite occurred for symptom outcomes (ASD symptom severity and social adaptive functioning), as the EIBI group had clearly greater symptom improvement than the other interventions group (g_c_ = −1.27, 0.65 vs. g_c_ = −0.03, 0.19). Additionally, stratified analyses could not be conducted in meta-analysis II because of the limitations of the controlled studies.

### Sensitivity Analysis

Sensitivity analyses suggested that the estimates were not substantially modified by any single study. There was an exception for the adaptive composite score, as a small effect with a g_c_ of 0.31 (95% CI 0.002 to 0.62) was shown when Magiati et al. (20) was removed in meta-analysis I. The sensitivity analyses did not yield different findings after the data were reanalyzed either using a fixed effects model or restricting to the between-group studies (see Supplementary Figures 1, 2 for the latter).

### Publication Bias

No sign of publication bias was found in the funnel plots and Egger's test for any outcome.

### Meta-Regression

**Figure 5:**
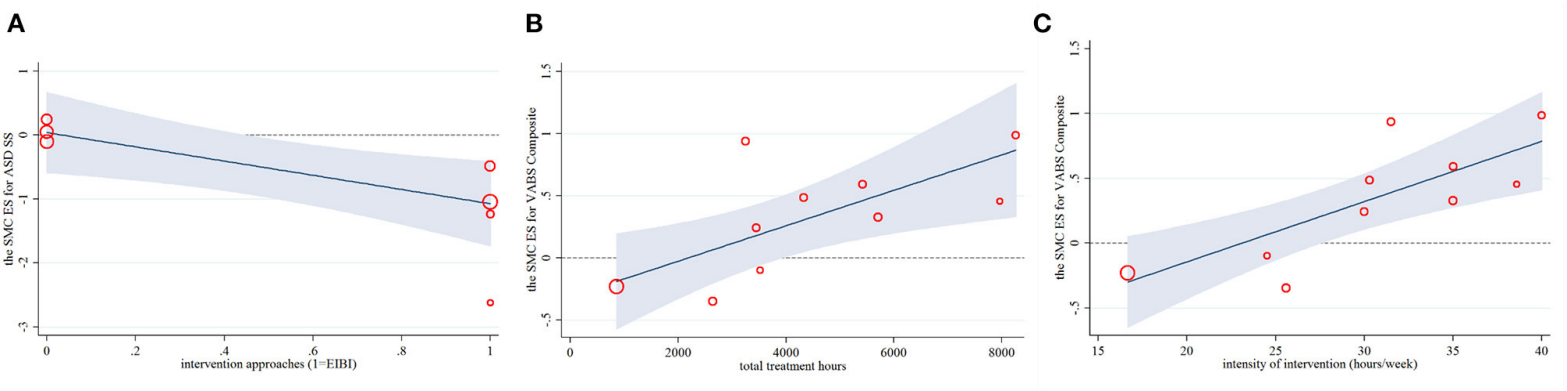
Regression plots for the primary intervention characteristics versus the ES for ASD symptom severity **(A)** and VABS composite **(B,C)**. ASD SS, ASD symptom severity; ES, effect size; SMC, standardized mean change; VABS, Vineland Adaptive Behavioral Scales.

**Figure 6:**
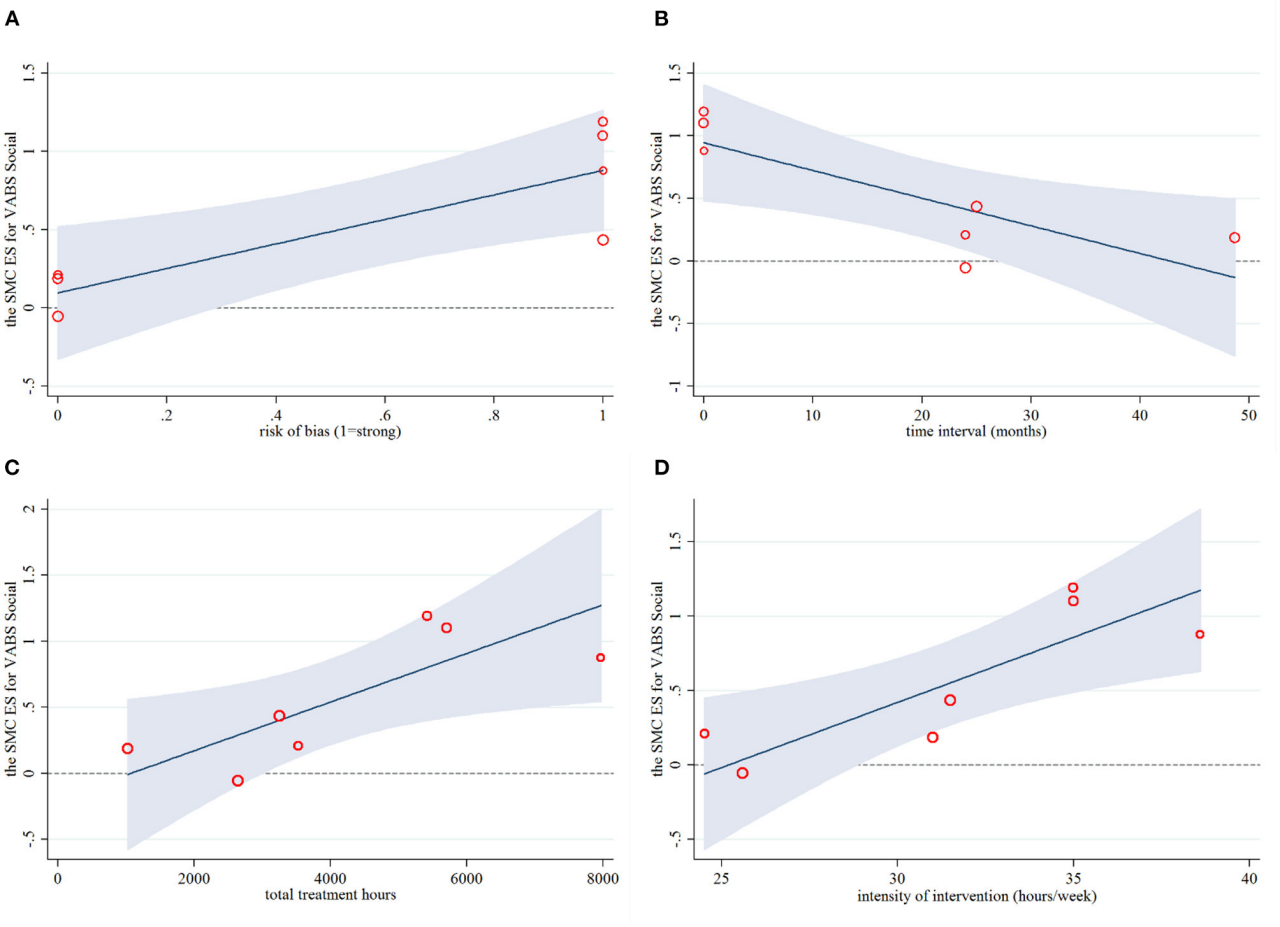
Regression plots for the primary intervention characteristics versus the ES for VABS social. ES, effect size; SMC, standardized mean change; VABS, Vineland Adaptive Behavioral Scales.

Across 11 predictors in univariate meta-regressions (Table 3), five mediators of longitudinal change in childhood outcomes emerged: (1) EIBI was more effective in reducing symptom severity than non-EIBI programs, and this explained 64% of the heterogeneity (Coefficient = −1.31, *P* = 0.045). (2) Higher total and social adaptive functioning were associated with longer total hours of the intervention explained 78 and 100% of the heterogeneity (Coefficient = 0.0001, *P* = 0.021; Coefficient = 0.0002, *P* = 0.032, respectively). Consistent results were found in the intensity of intervention, which both explained 100% of the heterogeneity (Coefficient = 0.047, *P* = 0.004; Coefficient = 0.087, *P* = 0.026, respectively). (3) Higher social adaptive functioning was also associated with a higher risk of bias (Adj R^2^ = 100.00%, Coefficient = 0.78, *P* = 0.026), and a shorter time interval between postintervention and follow-up (Adj R^2^ = 95.50%, Coefficient = −0.022, *P* = 0.033). In addition, the above results were verified by the regression plots, which displayed many significant linear correlations (see Figures 5, 6). No other confounding factors affected the change in the four outcome measures, and its regression plots were shown in Supplementary Figures 3–6 in the supplementary file.

**Table 3:** Results of the univariate meta-regression analyses by adaptation and symptomatic variables.

	**ASD SS**		**Composite^e^**		**DLS**		**Social**	
	**Coeff**	***P***	**Coeff**	***P***	**Coeff**	***P***	**Coeff**	***P***
**Internal Validity**								
Risk of bias^a^	1.100	0.33	0.450	0.16	0.019	0.97	**0.780**	0.03^*^
Sample size	0.020	0.41	−0.014	0.15	−0.037	0.63	−0.033	0.62
**Population Characteristics**								
Pre age	−0.080	0.17	−0.018	0.48	−0.046	0.46	−0.027	0.61
Pre IQ	−0.029	0.75	0.001	0.98	−0.014	0.74	0.011	0.83
Time interval^b^	−0.002	0.85	−0.002	0.65	0.009	0.53	**–0.022**	0.03^*^
Post age^c^	−0.005	0.64	−0.001	0.89	0.0006	0.98	−0.039	0.05
**Intervention Characteristics**								
Approaches^d^	**–1.310**	<0.05^*^	−0.704	0.18	−0.550	0.30	0.330	0.47
Total treatment hours	−0.0002	0.19	**0.0001**	0.02^*^	−0.0001	0.82	**0.0002**	0.03^*^
Intensity	−0.071	0.06	**0.047**	<0.01^*^	0.048	0.35	**0.087**	0.03^*^
Duration	−0.021	0.40	0.025	0.05	−0.014	0.50	0.026	0.10
Delivery agents	1.180	0.15	0.097	0.77	0.120	0.84	0.033	0.95

a *Categorical variable, strong = 1, non-strong (adequate and weak) = 0*.

b *Time interval between postintervention and follow-up*.

c *Mean age of participants at last assessment*.

d *Categorical variable, EIBI = 1, non-EIBI (ESDM and other interventions) = 0*.

e *Based on the result of sensitivity analysis, Magiati et al. (20) was removed from the meta-regression analyses*.

*ASD SS, ASD symptom severity; Coeff, unstandardized meta-regression coefficient; Composite, Vineland adaptive composite score; DLS, Daily living skills; Pre, preintervention*.

*ASD SS: Weiss and Delmolino (46); Smith et al. (43); Magiati et al. (20); Perry et al. (48); Vinen et al. (45); Clark et al. (36); Landa and Kalb (41)*.

*Composite: Sallow and Graupner (42); Howard et al. (40); Cohen et al. (37); Smith et al. (43); Kovshoff et al. (47); Weiss and Delmolino (46); Bibby et al. (35); Smith et al. (43); Perry et al. (48); Estes et al. (13)*.

*DLS and Social: Sallow and Graupner (42); Howard et al. (40); Cohen et al. (37); Smith et al. (44); Kovshoff et al. (47); Estes et al. (13); Akshoomoff et al. (34)*.

* *p < 0.05*.

*ASD symptom severity - Approaches: Adj R^2^ = 64.19%. Vineland adaptive composite score - Total treatment hours: Adj R^2^ = 78.06%. Vineland social adaptive score - Total treatment hours: Adj R^2^ = 100.00%. Vineland social adaptive score - risk of bias: Adj R^2^ = 100.00%. The bold values represents the p value < 0.05*.

The multivariate meta-regressions demonstrated a clear effect of implementer (therapist or therapist and parents) on IQ after the *p*-value was adjusted (*P* = 0.028, Table 4). Specifically, the involvement of parents in implementing intervention strategies had a more beneficial effect on IQ enhancement than the involvement of a therapist alone.

**Table 4:** Results of the multivariate meta-regression analyses by cognitive function.

	**Coefficient**	**SE**	**95% CI**	***P***	**tau^**2**^**	**k**	**Adj R^**2**^ (%)**	**Model *P***	**Type I errors^a^**
**IQ**									
Delivery agents^b^	0.6756	0.2637	[0.0881, 1.2632]	0.028^*^					
Pre age	−0.0289	0.0204	[−0.0742, 0.0165]	0.187	0.1294	14	52.15	0.048^*^	not
Total treatment hours	0.00000184	0.000046	[−0.0001, 0.0001]	0.969					

a *Monte Carlo permutation test was applied to correct type I errors for multiple covariate meta-regressions*.

b *Categorical variable: therapist = 1, therapist + parents = 2*.

*CI, confidence interval; Coefficient, unstandardized meta-regression coefficient; CTM, comprehensive treatment model; IQ, intelligence quotient; k, number of studies or “clusters”; Pre, preintervention; SE, standard error*.

*IQ: Sallow and Graupner (42); Howard et al. (40); Cohen et al. (37); McEachin et al. (33); Smith et al. (43); Kovshoff et al. (47); Smith et al. (44); Harris and Handleman (39); Bibby et al. (35); Magiati et al. (20); Estes et al. (13); Vinen et al. (45); Clark et al. (36); Gabriels et al. (38); Landa and Kalb (41)*.

* *p < 0.05. The bold values represents the p value < 0.05*.

## Discussion

To the best of our knowledge, this is the first comprehensive study to systematically and quantitatively assess a series of developmental and symptom outcomes for children with ASD. Overall, we found positive effects of early CTMs on longitudinal changes in intelligence, language development, communication and social adaptation, and core symptom severity in children with ASD but negligible effects on DLS and total adaptive behavior. In addition, there is preliminary evidence to suggest that children in the EIBI group have made greater gains than children in the control group with respect to intelligence, communication, and social adaptation. It is noteworthy that the outcomes and the risk of bias in most of the included studies are not optimistic. Nevertheless, we demonstrated that the treatment characteristics played a major role in the later outcomes for children younger than 5 years of age, which may also apply to some novel interventions.

The findings from this study are similar to those of a narrative review that examined the long-term effects of early intervention (EI) in primary school (15). The review included eight eligible studies, five of which were also included in our study. Both this review and the narrative review indicate that most children with ASD who have ever participated in a CTM make gains in many areas of functioning. However, only nine long-term follow-up studies were found based on our inclusion and exclusion criteria. In other words, the number of well-designed longitudinal studies is still insufficient to determine the long-term effects; therefore, more emphasis should be placed on empirical studies in this field in the future.

Although, favorable effects were apparent across most outcomes, language-related outcomes (IQ, receptive language, expressive language, and communication adaptation) were distinctly superior to social adaptation and ASD symptom severity, with ESs approaching 1.2 for receptive and expressive language. This finding is highly consistent with previous findings from a meta-analysis on the effects of ABA intervention in early childhood that included studies with a minimum intervention duration of 1 year (49) and has been attributed to the amount of time devoted by most behavioral interventions to language and communication skills (50).

In addition, there is some evidence that EIBI leads to a small to moderate effect in youth with ASD compared to the effect of treatment as usual, EIBI parent-mediated or EIBI minimal treatment controls in terms of IQ and Vineland social, communication, and adaptive composite scores. This is particularly noteworthy because these ESs were smaller than those from a Cochrane Collaboration systematic review and meta-analysis of studies comparing EIBI to treatment as usual in the community (51), which found medium to large significant positive effects. The comparison types of the controlled studies varied across the included studies, with nearly half of them involving implementer comparison (therapist vs. therapist and parents); stratification by comparison type was impossible due to the very small number of studies. Actually, the available evidence has proven the effectiveness of parent-mediated EI, showing improvement comparable with that achieved with therapist-mediated EI (52). Needless to say, the existence of this comparison type would weaken the ES.

It is generally believed that children participating in early CTMs will have a reduced need for support and programs as they go through school (47), but our study highlighted that despite some improvements, the outcomes of children with ASD are still far from normal. Thus, ongoing intervention is necessary, especially for adaptive functioning in real life. Even so, almost 30% of US children with ASD did not receive behavioral or medication treatment (53), and multiple gaps were identified across all the stages of intervention development and testing from conceptualization to community implementation (54). These may be crucial issues to fill to improve outcomes for individuals with ASD in the future.

Furthermore, a systematic review (19) of outcomes in late adolescence and adulthood was selected for comparison with our results to draw more reliable conclusions, and improvements in language and symptom outcomes were found in both children and adult populations. Our results, however, showed a significant gain in IQ and negative findings for adaptive functioning and DLS. Analyses of the distinctiveness of developmental trajectories with respect to these outcomes provided evidence of steady and remarkable improvements in verbal and non-verbal IQ from childhood to adolescence when the pre-IQ range in the included studies was 50–60 (55). Similarly, individuals with moderate adaptive functioning at baseline (standard score of ~75) had a stable trajectory (56). These findings suggest that longitudinal change could be influenced somewhat by the baseline level of participants, and our result explains the prognosis of ASD children with moderate functioning in terms of IQ and adaptation at baseline. Viewed from another angle, we did not find enough studies reporting the prognosis of lower- and higher-functioning ASD. Regarding the negative findings for DLS, Di Rezze et al. (57) indicated that an improvement in trajectory was associated only with lower and improving ASD symptom severity, whereas, none of the seven studies reported symptom-related data. We did not find any statistically significant population characteristics in the meta-regression, probably because the mean values of preintervention population variables were relatively concentrated among our included studies. Therefore, we propose that developmental and symptom outcomes could affect each other over time, and the effectiveness of CTMs should be examined by controlled studies designed for multiple subpopulations. Furthermore, the environmental factors that may be associated with continued changes in those outcomes from childhood to adulthood remain largely unknown (58) and may be responsible for the difference in the results.

Due to the variation in changes in childhood, we sought to explore the sources. Although, the ESDM was the most effective in improving IQ and EIBI showed greater efficacy in ASD symptom severity reduction in affected children, we are still far from establishing an evidence basis for the superiority or inferiority of the ESDM program because of the limited number of appropriately designed relevant studies. However, meta-regression provided a clear account of the impact of the implementer and intervention approach and verified the results of the stratified analyses: (1) IQ tended to benefit more from intervention programs mediated by parents and therapists, while the ESDM is an intervention strategy with parental involvement; (2) symptoms tended to benefit more from EIBI programs than non-EIBI programs. We did explore whether the quality and sample size of the studies, initial IQ or age of participants were related to deterioration/improvement in all outcomes over time. Only five significant associations were identified: intervention approach, implementer, total treatment hours, intervention intensity, and risk of bias; these derive almost entirely from intervention elements. Makrygianni et al. (23) have also suggested that the program intensity and duration are important predictors of the effectiveness of treatment on adaptive functioning. Thus, insufficient treatment time may account for the negligible effects on adaptive behavior.

### Limitations

The conclusions of this review should be interpreted with caution in light of its limitations. First, very few high-quality studies have specifically examined outcomes in childhood, and the numerous methodological weaknesses of the studies reviewed here limit the conclusions that can be drawn. Given that the studies varied widely in terms of cohort selection, treatment features, and assessment reliability, we could not establish an unbiased way of taking into account all these factors in judging research quality. We strongly endorse the conclusions of some reviews that rated the overall quality of evidence as “low” or “very low” using the GRADE system (7). Nevertheless, according to the current quality assessment, the quality level necessary to perform meta-regression was met, and most of the changes in the outcomes have nothing to do with the quality. Unfortunately, the LEAP program (59), which has a rigorous research-based design, was excluded from this review because of insufficient initial data.

Second, to achieve a certain statistical power, this study combined single-group prepost studies with between-group controlled studies to analyze the ES, although, this approach is somewhat controversial. However, similar results were obtained when we performed the meta-analysis II among the between-group studies only, indicating the reliability of our results.

Third, we used the group average age data as one of inclusion criteria due to a lack of individual raw data; therefore, it is inevitable that some children were preschoolers at follow-up and some were in their late teens. However, our results showed that the age at the last assessment did not affect the gains. We are looking forward to a time when investigators are willing to share their unpublished data, allowing meta-analyses on this topic to be more complete.

Finally, fidelity measures and standards cannot currently be assumed for studies in this field, and most did not provide information about additional treatment received after the intervention services ended.

### Recommendations for Future Research

In sum, recommendations for clinicians and researchers planning to conduct empirical studies in this area include the following: (1) employ study designs that use randomized controlled trials whenever possible and match treatment intensity and duration across groups; (2) record the specific intervention approaches and components in detail and monitor the fidelity of the intervention process; (3) collect detailed information on education and intervention strategies applied during mid-childhood and adolescence; (4) due to the current need, explore ESDM programs and lower- and higher-functioning ASD; and (5) focus on follow-up measurement and record the initial measurement as comprehensively as possible.

## Conclusion

Overall, there is some evidence that most children with ASD who participate in an early CTM make gains in many areas of functioning, especially with respect to symptom- and language-related outcomes. However, most of the existing research relies on small studies that are non-randomized, forestalling definitive conclusions. What is certain is that the childhood outcomes of children with ASD are still far from normal, especially with respect to adaptive functioning, and the mediating variables of developmental gains were primarily intervention elements, including approach, implementer, intensity, and total treatment hours. Furthermore, the ESDM displayed the largest effect in terms of improving intelligence development, and EIBI showed greater efficacy in reducing ASD symptom severity.

## Data Availability Statement

The raw data supporting the conclusions of this article will be made available by the authors, without undue reservation.

## Author Contributions

BS, LC, and JJ were involved in the conception and design of the review. BS, WW, and MD contributed to the data collection. JZ and JL contributed to the quality assessment. BS and JZ conducted the meta-analyses. BS and MD contributed to interpretation of data. The review was conducted by BS and WW, who completed initial drafts of the paper. LC, BW, and JJ gave critical comments and advice that helped shape the review. All authors read and approved the final manuscript.

## Conflict of Interest

The authors declare that the research was conducted in the absence of any commercial or financial relationships that could be construed as a potential conflict of interest.

## Publisher's Note

All claims expressed in this article are solely those of the authors and do not necessarily represent those of their affiliated organizations, or those of the publisher, the editors and the reviewers. Any product that may be evaluated in this article, or claim that may be made by its manufacturer, is not guaranteed or endorsed by the publisher.

## Abbreviations

ABA: Applied Behavior Analysis
ASD: Autism Spectrum Disorder
CI: Confidence Interval
CTM: Comprehensive Treatment Model
DLS: Daily Living Skills
DSM: The Diagnostic and Statistical Manual of Mental Disorders
EIBI: Early Intensive Behavioral Intervention
ES: Effect Sizes
ESDM: Early Start Denver Model
FIP: Focused Intervention Practices
GRADE: Working Group Grades of Evidence
IQ: Intelligence Quotient
JASPER, Joint Attention: Symbolic Play and Engagement Regulation
LEAP: Learning Experiences - An Alternative Program for Preschoolers and Parents
MeSH: Medical Subject Headings
NICE: National Institute for Health and Care Excellence
PACT: Pre-school Autism Communication Trial
RCT: Randomized Controlled Trial
SMD: Standardized Mean Difference
UCLA, University of California: Los Angeles
VABS: Vineland Adaptive Behavioral Scales.
