# Supplementary material for: Cognitive, Language, and Behavioral Outcomes in Children With Autism Spectrum Disorders Exposed to Early Comprehensive Treatment Models: A Meta-Analysis and Meta-Regression
Source: Front Psychiatry. 2021 Jul 26;12:691148. doi: 10.3389/fpsyt.2021.691148 (PMC8350444; doi:10.3389/fpsyt.2021.691148)
Supplement: Supplementary file 1 [file Data_Sheet_1.docx]

**Table S1**. Study inclusion and search strategy.

|  | Criteria | Search terms |
| --- | --- | --- |
| Participants | Autism spectrum disorder, middle children (6-to-18-year-old) | (autis* OR ASD$ OR Asperger OR PDD-NOS) AND (adolescen* OR youth OR teenage OR young OR juvenile OR children OR school-age OR middle NOT adult) |
| Intervention | Behaviour/developmental, Comprehensive treatment models, No pharma component | (intervent* OR " comprehensive treatment models " OR "EIBI" OR "ESDM" OR "TEEACH" OR "LEAP") |
| Comparison | Any comparison design study |  |
| Outcomes | Any developmental or sympto-matic outcomes | (outcome OR prognosis OR predict OR change OR trajectory) |
| Study design | Any group design study |  |
| Other | English journals,humans research | La.exact(English & humans) |

**Table S2**. Outcomes and measurement of the included studies.

| Outcome | Study | Measurement |
| --- | --- | --- |
| IQ | b, c, d, e, f, g, h, i, j, k, l, n, o, p, q | WISC-II, WISC-III, WISC-R, BSID, BSID-R, BSID-II, WPPSI, WPPSI-R, Merrill-palmer, MSEL, WASI, DAS,S-B, DP-II, Leiter-R. |
| Expressive Language | d, h, l, n, o | Reynell, SICD-R, EOWPVT, BPVS-2. |
| Receptive Language | d, h, l, n, o | Reynell, SICD-R, EOWPVT, BPVS-2. |
| ASD Symptom Severity | c, j, l, m, p, q, r | ADOS-G, ADOS; ADI-R, CARS. |
| VABS composite | b, d, e, h, i, l, m, n, o, p, r | VABS, VABS-II, VABS-Survey. |
| VABS communication | a, d, e, h, i, j, n, o | VABS, VABS-II, VABS-Survey. |
| VABS social | a, d, e, h, i, n, o | VABS, VABS-II, VABS-Survey. |
| VABS daily living skills | a, d, e, h, i, n, o | VABS, VABS-II, VABS-Survey. |

Key: a—Akshoomoff et al (2010); b—Bibby et al (2002); c—Clark et al (2017); d—Cohen et al (2006); e—Estes et al (2015); f—Gabriels et al (2001); g—Harris et al (2000); h—Howard et al (2014); i—Kovshoff et al (2011); j—Landa and Kalb (2012); k—McEachin et al (1993); l—Magiati et al (2011); m—Perry et al (2017); n—Sallows et al (2005); o—Smith et al (2000); p—Smith et al (2015); q—Vinen et al (2017); r—Weiss and Delmolino (2006).

ADOS, the Autism Diagnostic Observation Schedule; ADI-R, the Autism Diagnostic Interview, Revised; BSID, the Bayley Scales of Infant Development; BPVS, the British Picture Vocabulary Scales; CARS, Childhood Autism Rating Scale; CELF III, Clinical Evaluation of Language Fundamentals, Third Edition; Com, communication; DAS, the Differential Ability Scales; DLS, daily living skills; DP-II, Developmental Profile-II; DQ, developmental quotient; ELC, the early learning composite; EOWPVT, the Expressive One-Word Picture Vocabulary Test; GCA, general conceptual ability score; IQ, intelligence quotient; MSEL, Mullen Scales of Early Learning; PLS-III, the preschool language scale-third edition; RBS-R, the Repetitive Behavior Scale-Revised; REEL, the Receptive-Expressive Emergent Language Scale; Reynell, the Reynell Developmental Language Scales; RRB, restricted and repetitive behavior; SA, social affect; S-B, the Stanford-Binet Intelligence Scale; SI, social interaction; SICD-R, the Sequenced Inventory of Communication Development -Revised; Soc, socialization; WASI, the Wechsler Abbreviated Scales of Intelligence; WISC, the Wechsler Intelligence Scale for Children; WPPSI, the Wechsler Preschool and Primary Scales of Intelligence; VABS, Vineland Adaptive Behavior Scales.

**Table S3**. Quality indicators identified in comprehension literature.

| Indicator | Akshoomoff | Bibby | Clark | Cohen | Estes | Gabriels | Harris | Howard | Kovs  Hoff | Landa | McEachin | Magiati | Perry | Sallows | Smith 2000 | Smith  2015 | Vinen | Weiss |
| --- | --- | --- | --- | --- | --- | --- | --- | --- | --- | --- | --- | --- | --- | --- | --- | --- | --- | --- |
| *Primary indicators* |  |  |  |  |  |  |  |  |  |  |  |  |  |  |  |  |  |  |
| Participants | H | A | H | H | H | A | H | H | A | H | H | A | H | H | A | H | H | H |
| Independent variable | H | A | U | H | H | A | H | H | H | A | H | H | H | H | H | H | H | H |
| Comparison condition | U | U | U | H | H | U | U | H | H | U | H | U | U | H | H | U | H | U |
| Dependent variable | H | H | H | H | H | H | A | H | H | H | H | H | H | H | H | H | H | H |
| Link ^ⱡ^ | H | H | H | H | H | H | H | H | H | H | H | H | H | H | H | H | H | H |
| Statistical analysis | H | H | H | H | H | H | H | H | H | H | H | H | H | H | H | H | H | H |
| *Secondary indicators* |  |  |  |  |  |  |  |  |  |  |  |  |  |  |  |  |  |  |
| Random assignment | - | - | - | - | + | - | - | - | - | - | - | - | - | + | + | - | - | - |
| Interobserver agreement | - | + | + | - | - | - | - | - | + | - | + | + | - | + | + | + | - | + |
| Blinding of raters | + | + | + | + | + | + | - | + | - | + | + | + | + | + | + | - | + | - |
| Fidelity | - | - | - | + | + | - | + | + | + | + | + | + | - | + | + | + | + | - |
| Attrition | - | - | - | + | + | - | - | + | + | - | - | - | - | + | + | - | + | - |
| Generalization or maintenance | + | - | - | - | + | - | + | - | + | + | + | + | + | - | + | + | - | - |
| Effect size | + | + | + | + | + | + | + | - | + | + | + | + | + | + | - | + | + | + |
| Social validity | + | + | + | + | + | + | + | + | + | + | + | + | + | + | + | + | + | + |
| *Rating* | W | W | W | S | S | W | W | S | A | W | S | W | W | S | A | W | S | W |

ⱡ link between research question and data analysis.

Abbreviations: A, Acceptable; H, High; U, Unacceptable.

Rating: A, Adequate; S, Strong; W, Weak.

Note: **Participant characteristics:** Age and gender were provided for all participants, specific diagnostic information was provided for all participants with autism, if applicable, standardized test scores were provided, and information on the characteristics of the interventionist was provided. **Independent variable:** Information about the treatment was provided with replicable precision (if a manual was used, this was always given a high quality rating). **Comparison condition:** The conditions for the comparison group were defined with replicable precision, including, at a minimum, a description of any other interventions participants received. **Dependent variable:** Dependent measures were described with operational and replicable precision, showed a clear link to the treatment outcome, and were collected at appropriate times. **Link between research question and data analysis:** Data analyses were strongly linked to the research question(s) and the data analysis used correct units of measure (i.e., child level, teacher level, etc.) on all variables.

Use of statistical tests: Proper statistical analyses were conducted for each statistical measure with an adequate power and a sample size of n≥10. **Random assignment:** Participants were assigned to groups using a random assignment procedure. **Interobserver agreement:** IOA was collected across all conditions, raters, and participants with inter-rater agreement at or above 0.80, and a minimum of Good reliability (k≥0.60). Psychometric properties of standardized tests were reported and were equal or greater than 0.70 agreement with a k≥0.40. **Blind raters:** Raters were blind to the treatment condition of the participants. **Fidelity:** Procedural fidelity or treatment fidelity was continuously assessed across participants, conditions, and implementers, and if applicable, had measurement statistics at or greater than 0.80. **Attrition:** Articulation was comparable (did not differ between groups by more than 25%) across conditions and less than 30% at the final outcome measure. **Generalization and/or maintenance:** Outcome measures were collected after the final data collection to assess generalization and/or maintenance. **Effect size:** Effect sizes were reported for at least 75% of the outcome measures and were equal or greater than 0.40. **Social validity:** The study contained at least four of the following; (a) DVs were socially important (i.e., would society value the changes in outcome of the study), the (b) intervention was time and cost effective (i.e., did the ends justify the means), (c) comparisons were made between individuals with and without disabilities, (d) the behavioral change was large enough for practical value (clinically significant), (e) consumers were satisfied with the results, (f) people who typically come in contact with the participant manipulated the IVs, (g) the study occurred in natural contexts.”


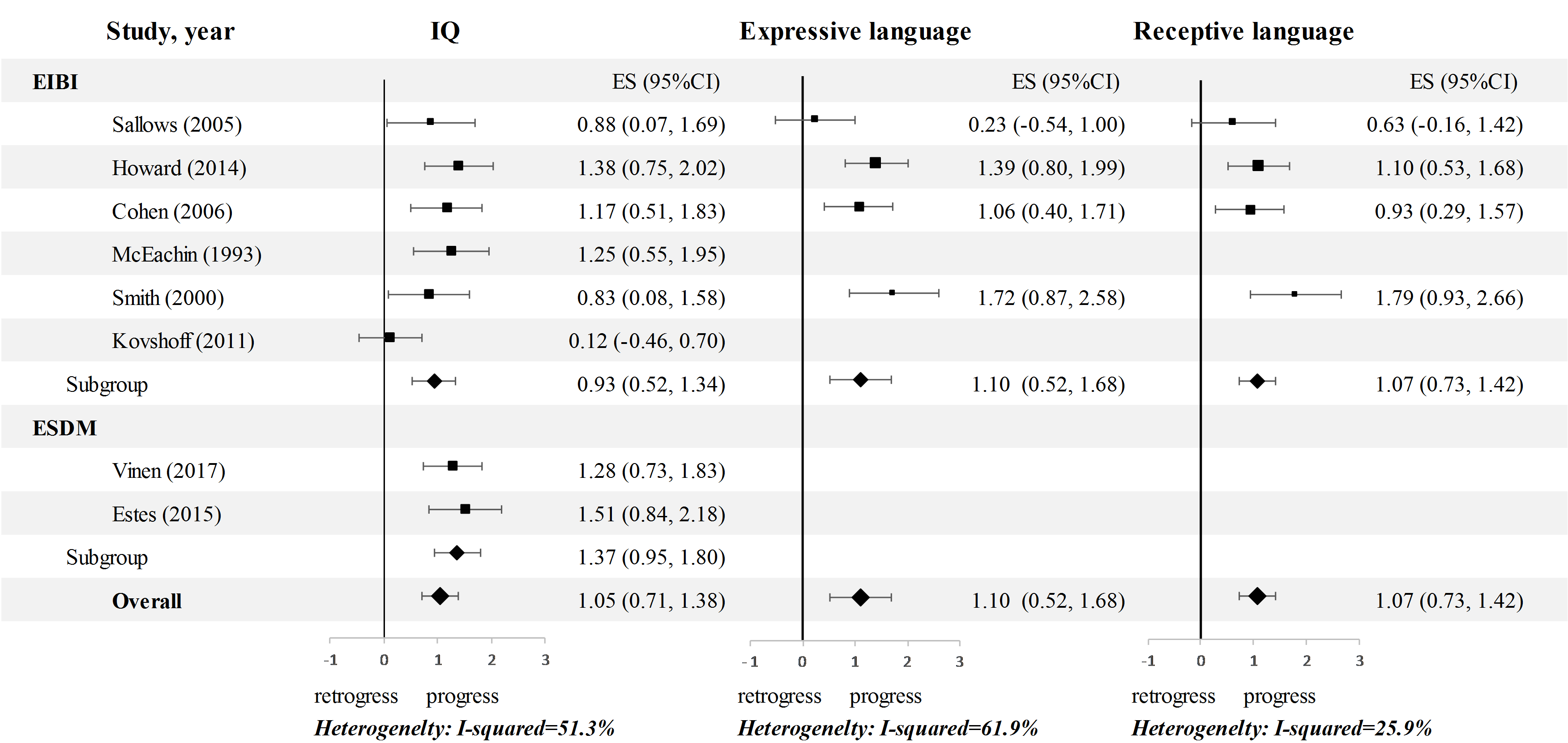


Fig S1. Re-Meta-analysis I: Effect sizes (g_c_) for IQ composite and language of early intervention, which were pooled from the between-group controlled studies.


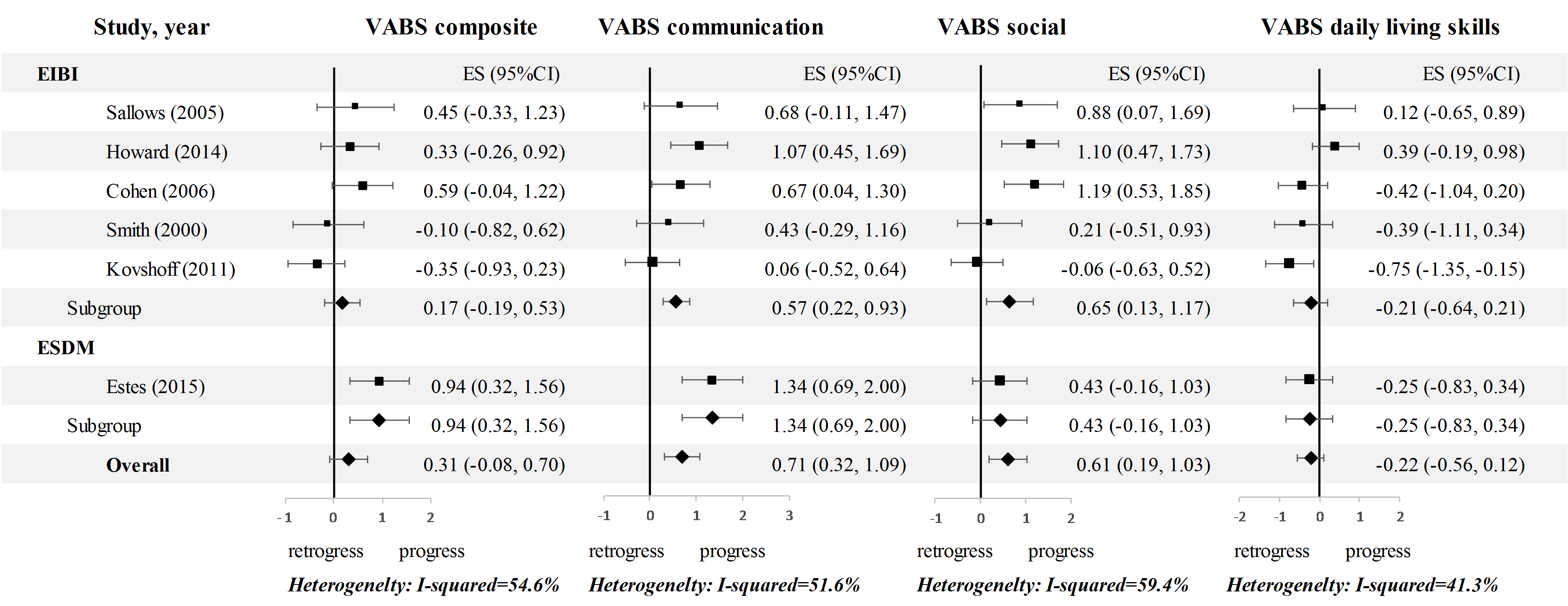


Fig S2. Re-Meta-analysis I: Effect sizes (g_c_) for adaptive behavior of early intervention, which were pooled from the between-group controlled studies.


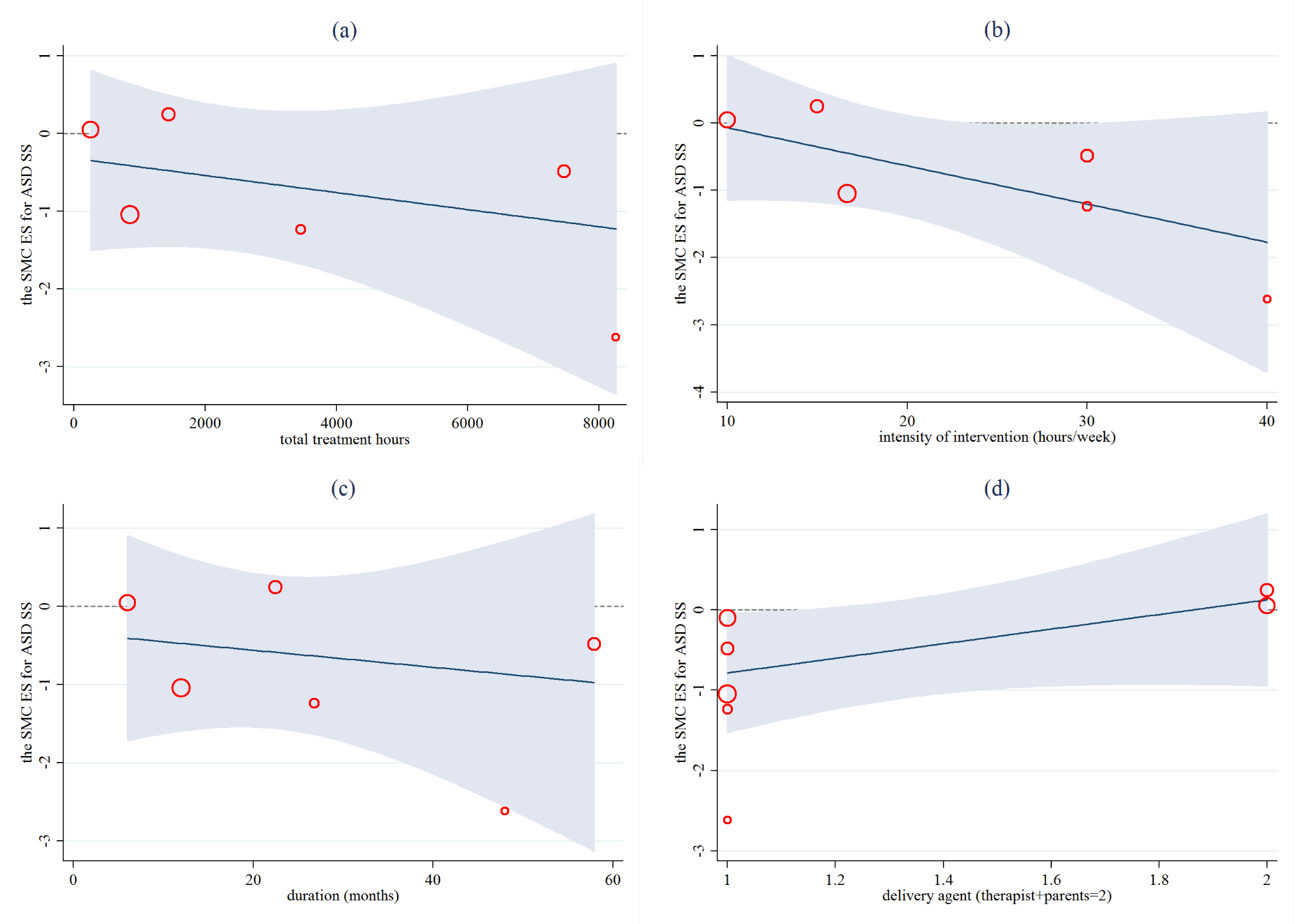


Fig S3. Regression plots for the remaining intervention characteristics versus the ES for ASD symptom severity.


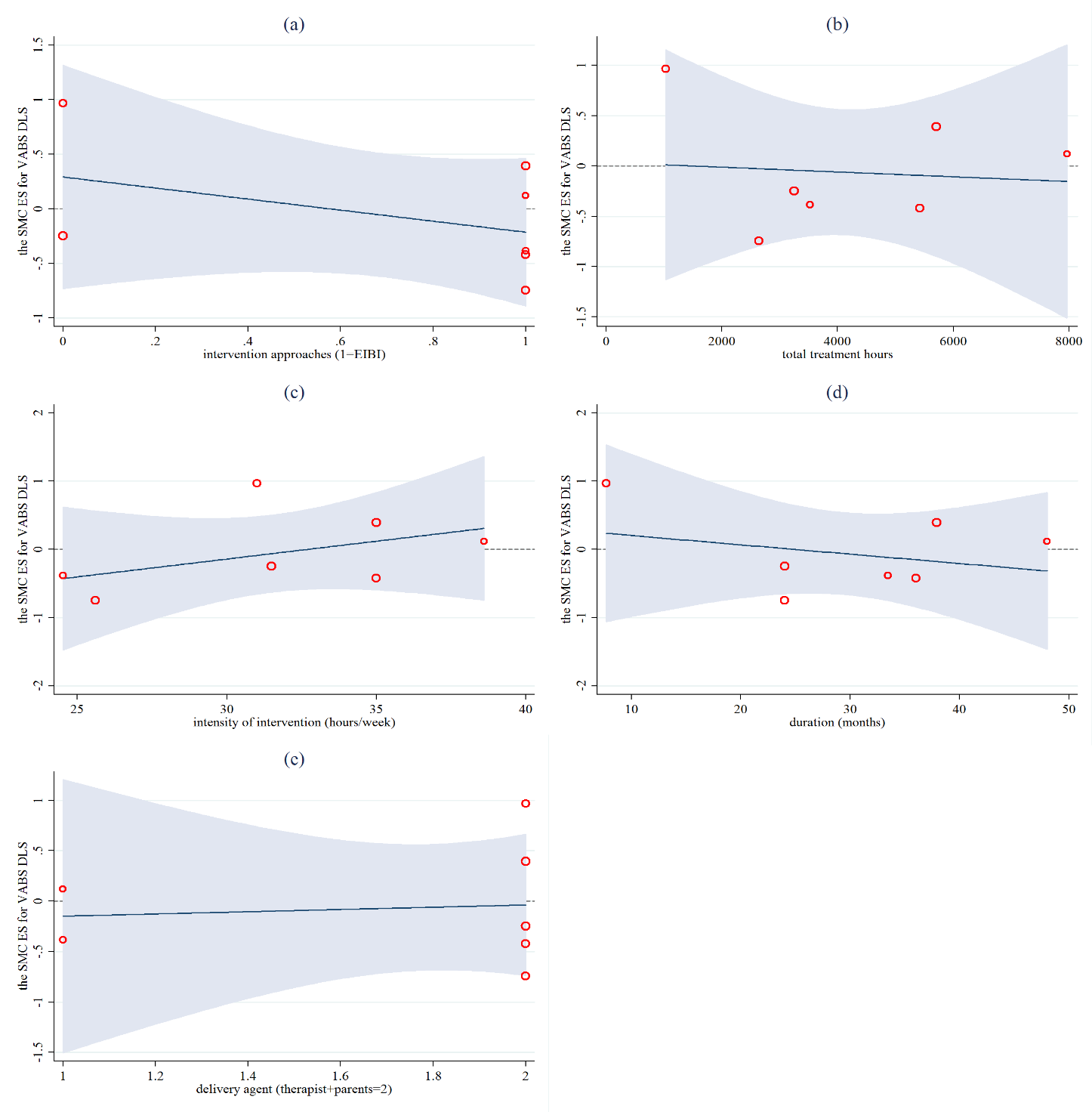


Fig S4. Regression plots for the intervention characteristics versus the ES for VABS DLS.


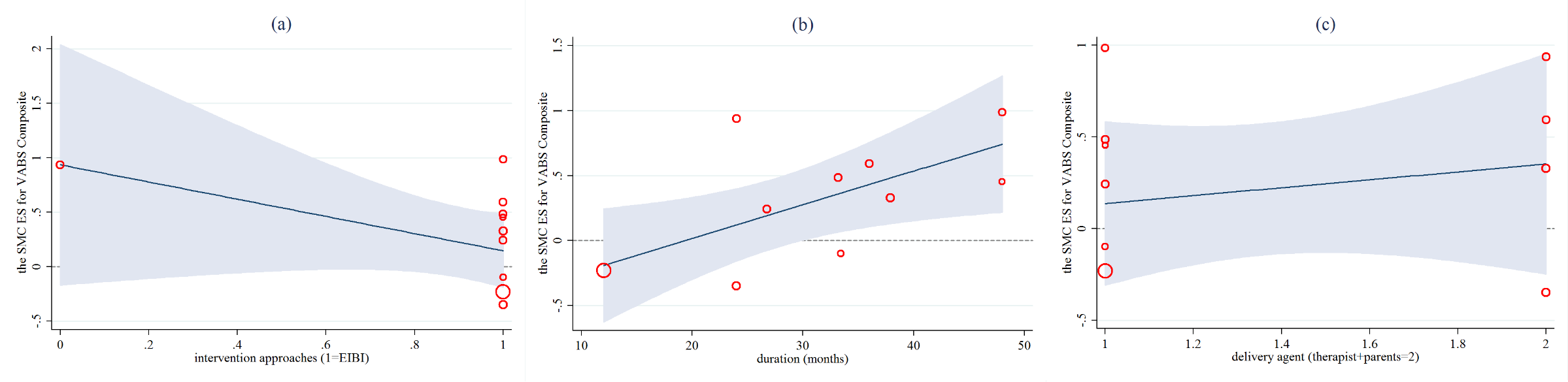


Fig S5. Regression plots for the remaining intervention characteristics versus the ES for VABS composite.


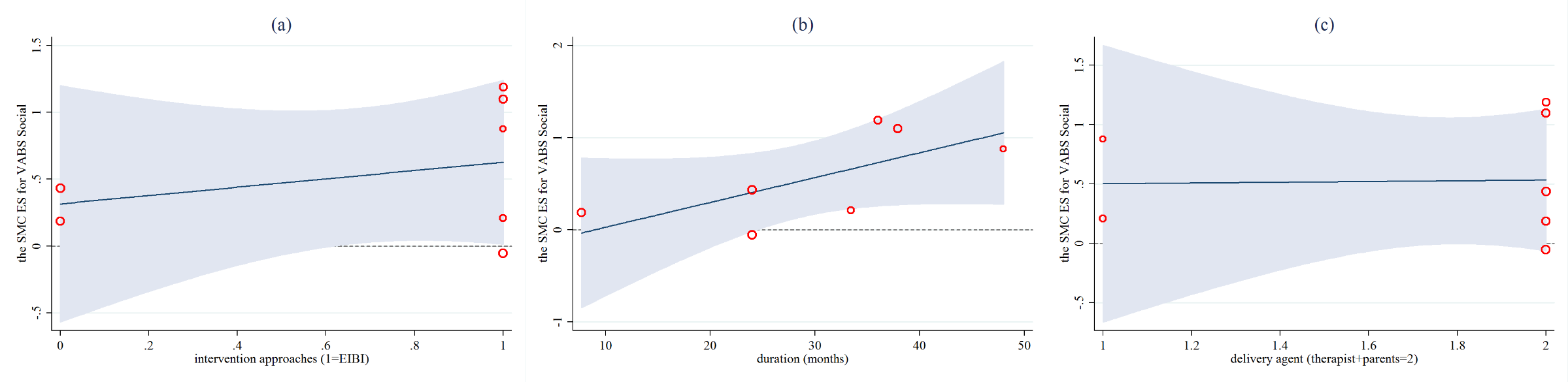


Fig S6. Regression plots for the remaining intervention characteristics versus the ES for VABS social.
